# Integrin VLA-5 and FAK are Good Targets to Improve Treatment Response in the Philadelphia Chromosome Positive Acute Lymphoblastic Leukemia

**DOI:** 10.3389/fonc.2014.00112

**Published:** 2014-05-15

**Authors:** Zhongbo Hu, William B. Slayton

**Affiliations:** ^1^Division of Hematology and Oncology, Department of Pediatrics, University of Florida, Gainesville, FL, USA

**Keywords:** integrin α5β1, focal adhesion kinase, acute lymphoblastic leukemia, Philadelphia chromosome, TAE226, bone marrow microenvironment, tyrosine kinase inhibitor

## Abstract

Acute lymphoblastic leukemia bearing the Philadelphia chromosome is among the most difficult types of ALL to cure. However, the advent of targeted tyrosine kinase inhibitor (TKI) imatinib has ushered in a new era of treatments that have the potential to be less toxic to patients. Integrins and tyrosine kinases play important roles in mediating and transducing signals for cell survival and suppressing apoptosis. Focal adhesion kinase (FAK) is a non-receptor type tyrosine kinase that is constitutively activated in Ph+ ALL. We sought to investigate the specificity of integrin α5β1 (VLA-5) on Ph+ leukemia by its expression and function. We found VLA-5 expression increases after serum starvation. Integrin α5 inhibitory antibody inhibited adhesion of Ph+ leukemia to human fibronectin and acted synergistically with imatinib to induce Ph+ leukemia cell apoptosis. We used different strategies to block integrin signaling and knocked down the expression of integrin VLA-5 to observe the effect on proliferation and engraftment of Ph+ leukemia cells in immunodeficient mice. We found that blocking integrin activity by incubating Ph+ leukemia cells with disintegrin, a peptide inhibitor of integrins, or α5 inhibitory antibody, or knocking down the α5 integrin subunit impaired and delayed the engraftment of Ph+ leukemia in immunodeficient mice. We then treated mice xenografted with Ph+ leukemia cells with the FAK inhibitor TAE226 in combination with a BCR–ABL TKI nilotinib. While 2 weeks of treatment with TAE226 alone did not significantly inhibit leukemia growth in mice, TAE226 in combination with nilotinib provided the most optimum growth inhibition at 4–6 weeks. We conclude that blocking VLA-5 signaling or combining FAK inhibitors with TKI targeting BCL/ABL might be good strategies to improve treatments in patients with Ph+ ALL. By altering Ph+ leukemia cell interactions with the microenvironment, we may increase their susceptibility to therapy targeting BCR/ABL.

## Introduction

Acute lymphoblastic leukemia bearing the Philadelphia chromosome (Ph+ ALL) is among the most difficult types of ALL to cure. BCR–ABL tyrosine kinase inhibitors (TKIs) such as imatinib, nilotinib, or dasatinib, which target the leukemogenic tyrosine kinase related to Philadelphia chromosome, are less effective in the treatment of Ph+ ALL than chronic myelogenous leukemia when used as single agents (1). Many lines of evidence suggest that interfering with the protective effect of bone marrow microenvironment in combination with BCR–ABL TKIs could be of benefit for the eradication of the leukemic cells.

Integrins, a family of adhesion molecules, and tyrosine kinases play important roles in mediating and transducing signals for cell survival and suppressing apoptosis. Integrins are heterodimeric signaling and adhesion molecules consisting of an alpha and beta chain. Ligands for these receptor proteins, such as collagens, fibronectins, and laminins, and cellular receptors, such as vascular cell adhesion molecule-1 (VCAM-1) and the intercellular cell adhesion molecule (ICAM) family bind integrins and mediate a variety of cell-matrix and cell–cell adhesion functions, affecting endothelial cells, leukocytes, and leukemia or other tumor cells behavior. Integrin α5β1, also known as very late activation antigen-5 or VLA-5 promotes cell survival through interaction with the extracellular matrix by activating Bcl-2, migration through activation of RhoA, and proliferation through activation of ERK, Akt, and FAK (2). Integrin α5β1 specifically mediates the survival and apoptosis resistance of acute lymphoblastic leukemia cells with the Philadelphia chromosome (3–5).

Focal adhesion kinase (FAK) is a non-receptor type tyrosine kinase that is constitutively activated in Ph+ ALL. FAK can be activated by integrins, and growth factor receptors (6). FAK signaling is critical for cell proliferation, differentiation, and apoptosis, and is an important determinant of tumor aggressiveness (7). Integrin signaling through FAK is required for certain cancer cells to grow (8). FAK activates and stabilizes the IGF-1 receptor, which is involved in the constitutive activation of PI3K leading to proliferation of leukemia cells (9). FAK has been shown to be activated in Ph+ ALL (10), and is therefore an attractive molecular target. FAK silencing inhibited leukemogenesis in BCR/ABL-transformed hematopoietic cells (11). This has made FAK inhibitors interesting candidates for B cell acute lymphoblastic leukemia treatment.

We hypothesized that survival of Ph+ leukemia cells can be inhibited by reducing signaling through VLA-5 or FAK. We test the possibility that by interfering with VLA-5 and FAK signaling, we will increase the susceptibility of Ph+ ALL cells to therapy targeting BCR/ABL.

## Materials and Methods

### Cell culture

The human bone marrow stromal cell line, HS-5, and the Philadelphia chromosome positive B cell acute lymphoblastic leukemia cell line, SUP-B15, were purchased from ATCC. HS-5 was cultured in Dulbecco’s Modified Eagle’s Medium plus 10% fetal bovine serum. SUP-B15 was maintained in Iscove’s Modified Dulbecco’s Medium with 4 mM l-glutamine, 1.5 g/l sodium bicarbonate and supplemented with 0.05 mM 2-mercaptoethanol, 20% fetal bovine serum, and 50 U/ml penicillin/streptomycin. In order to be tracked *in vivo* in animals, SUP-B15 cells were infected with lentivirus-vector expressing system LV-luciferase (provided by Dr. Lung-Ji Chang, Department of Molecular Genetics and Microbiology, University of Florida) and selected for stabilized expressing clones by series dilution selection. The stabilized expressing luciferase cell line was renamed SUP-LUC2.

### Flow cytometry

The expression of integrin subunits on SUP-B15 cells before or after serum starvation was detected by flow cytometry. The expression level of integrin α5 after knocking down by integrin α5 shRNA lentiviral transduction was confirmed by flow cytometry. Cell apoptosis assay via PI combining with annexin-V were detected using BD LSR II flow cytometer and analyzed with FACSDiva software (BD Biosciences, San Jose, CA, USA). One million total cells per sample were analyzed. SUP-B15 was cultured on top of stromal cells HS-5 for 24 h compared without stromal cells. Ten micrograms per liter purified no azide/low endotoxin (NA/LE) mouse anti-human CD49e (clone: IIA1) or isotype IgG control and imatinib or vehicle control DMSO diluted with the same concentration in imatinib were used as different conditions. A high dose of imatinib of 10 μM was used because SUP-B15 cells were shown to be resistant to imatinib before with IC50 2 μM (12).

### Cell adhesion assay

Tissue culture-treated polystyrene 96-well microplates were coated with human fibronectin or fibronectin fragments at a concentration between 5 and 10 μg/ml on the day before use. Two hours prior to the assay, the fibronectin coated wells were aspirated and blocked with 2.5% bovine serum albumin (BSA) in phosphate buffer saline (PBS) for at least 2 h at room temperature or overnight at 4°C. Then, the microplates were washed with 100 μl PBS. Series dilution of different antibodies purified NA/LE mouse anti-human CD29 (clone HUTS-21), anti-human CD49d (Clone: 9F10), anti-human CD49e (clone: IIA1), and isotype IgG control with 100× of final concentrations was prepared in PBS with Ca++ and Mg++. 50 μl PBS with Ca++ and Mg++ with or without series diluted antibodies was added to each well. Prior to seeding, leukemia cells were stained with calcein AM (Invitrogen) at a final concentration of 1.25 μM for 30 min, washed, and activated with phorbol 12-myristate 13-acetate (Sigma) at 50 ng/ml for 7 min. Cells were washed immediately prior to plating. One hundred microliters of prepared cell suspension was added to each well in triplicate. The plates were centrifuged at 411 × *g* for 2 min to insure that the cells were in contact with the plate surface and incubated for 30 min at 37°C. The relative level of fluorescence of the samples prior to washing (Relative Fluorescence Units or RFU_before wash_) was measured using fluorescence Victor V microplate reader (Perkin Elmer) at excitation wavelength of 485 nm and emission wavelength of 520 nm. Then, the non-adherent cells were washed away with PBS twice and the wells were refilled with 100 μl PBS. The level of fluorescence after washing (RFU_after wash_) was measured using a plate reader. The percent of adherent cells was calculated using the following formula: [(RFU_after wash_ − RFU_background_)/(RFU_before wash_ − RFU_background_)] × 100. RFU_background_ is the RFU for wells lacking cells. The level of inhibition was calculated using the formula: inhibition (%) = 100 − 100 × percent adherent cells (treated)/percent adhesion of cells (without treatment).

### Cell proliferation assays

Cell proliferation was analyzed by MTT assay using the Cell Proliferation Kit I (MTT; Roche) as recommended by the manufacturer. SUP-B15 cells were seeded onto 96-well plates at a concentration of 2 × 10^4^ cells/well. TAE226 stock buffer was suspended in DMSO at 10 mM. A final concentration for TAE226 from 10 to 0.02 μM was prepared by serial dilution and added to the seeded cells with a final DMSO concentration of <0.1%. After 24, 48, and 72 h, cells were incubated for 4 h with 0.5 mg/ml MTT [3-(4,5-dimethylthiazol-2-yl)-2,5-diphenyltetrazolium bromide, a tetrazole] dye in complete cell culture medium. After solubilization of the purple formazan crystals, absorbance was measured at 570 nm (background wavelength, 650 nm) using a plate spectrophotometer. We used Prism to calculate the IC 50 dosage.

### Integrin α5 knock-down by shRNA lentiviral transduction

The spinning infection method was used to enhance the efficiency of lentiviral particle transduction. SUP-LUC2 cells growing in exponential phase were cultured overnight in fresh complete medium. The medium was replaced with a polybrene/media mixture at a final concentration of 5 μg/ml polybrene immediately before lentivirus infection. Human integrin α5 shRNA lentiviral particles (Catalog # sc-29372-V, Santa Cruz) were thawed at room temperature and added to leukemia cell suspension in 15 ml conical tubes and were spun at 800 × *g* (2500 rpm) for 90 min at 37°C. After spinning, the supernatant was aspirated and the cells were replenished in the fresh culture medium for ~48 h. Two days later, stable clones were selected using complete medium with puromycin. Two weeks later, leukemia cells were cultured with regular medium without puromycin. During selection, the medium containing puromycin was changed once a week.

In order to select stable clones expressing the shRNA to knock-down integrin α5 gene expression via puromycin dihydrochloride (sc-108071, Santa Cruz) selection, a titration of puromycin concentrations from 0 to 2.5 μg/ml were used to incubate SUP-LUC2 cells for culture over 2 weeks. After 1 week, the concentrations of puromycin to kill cells and the one in which cells were still alive were noted. At the 2 week time point, the wells with all dead cells were noted. The minimum level of puromycin dose, 0.8 μg/ml, which killed SUP-LUC2 cells by week 1 and in which there were no surviving cells/regrowth at 2 weeks, was chosen for selection.

### Quantitative reverse transcriptase-polymerase chain reaction

Total RNA was extracted by RNeasy Mini Kit (Qiagen, Valencia, CA, USA). The yield of purified RNA was determined by a spectrophotometer (NanoDrop 2000; Thermo Scientific, Wilmington, DE, USA). cDNA was prepared from 1.0 μg of total RNA with AffinityScript™ QPCR cDNA Synthesis Kit (Stratagene, La Jolla, CA, USA). QPCR amplification of cDNA was performed using the Brilliant^®^ II SYBR^®^ Green QPCR Master Mix with Stratagene Mx3000P. Primers for integrin α5 was purchased from Santa Cruz (Catalog # sc-29372-PR). GAPDH primers forward TGCACCACCAACTGCTTAGC and reverse GGCATGGACTGTGGTCATGAG were used as control. The cycle threshold (Ct) of the target gene (integrin α5) was normalized to the chosen reference gene GAPHD. Relative quantification, *R* = 2^−(ΔCt sample − ΔCt control)^. Result showed the fold change normalized to SUP-LUC2 cells.

### Western blotting

For Western blotting, 0.5 to 1 × 10^7^ cells were washed in cold PBS. Cells were lysed for 30 min in cell extraction buffer (Invitrogen, Camarillo, CA, USA) supplemented with 1 mM PMSF, protease inhibitor cocktail (Roche), and 1:100 diluted phosphatase inhibitor cocktail 2 (Sigma). Clear lysate was obtained by centrifugation at 13,000 rpm for 10 min at 4°C. Protein concentration was analyzed with Pierce™ BCA Protein Assay Kit (Pierce, Rockford, IL, USA). Fifty to hundred micrograms of protein were mixed with 6× SDS sample buffer and boiled for 10 min before loading. Proteins were resolved on polyacrylamide SDS gels (SDS-PAGE) and transferred to nitrocellulose (membrane Hybond-C super, Millipore). The membrane was blocked for 1 h at room temperature in Tris-buffered saline (TBS) containing 5% fat-free milk and then was probed overnight at 4°C overnight with the FAK, phospho-FAK (Tyr397) antibody, phospho-p44/42 MAPK (Erk1/2), and phospho-Akt (Thr308) antibodies (Cell Signaling), β-actin antibody (Santa Cruz), and mouse Anti-Human CD49e (BD Biosciences) at the concentration suggested by manufacturers in TBS, 0.1% Tween, 3% fat-free milk, and 3% BSA (Euromedex). After incubation for 1 h at room temperature with either anti-mouse or anti-rabbit IgG antibody coupled to horseradish peroxidase, detection was achieved using a chemiluminescent substrate (SuperSignal, Amersham Pharmacia Biotech).

### Animal studies

Mouse strains NOD/LtSz-scid/J (NOD/SCID) and NOD.Cg-*Prkdc^scid^* *Il2rg^tm1Wjl^*/SzJ, commonly known as NOD/SCID gamma mice (NSG), were bred and housed in the specific pathogenic-free (SPF) facility at the UF Health Cancer Center. The breeding pairs were purchased from Jackson Laboratories (Bar Harbor, Maine). These studies were approved by the University of Florida Institutional Animal Care and Use Committee.

#### Short term engraftment study with integrin α5 antibody in NOD/SCID mice

Before injection, SUP-B15 cells were incubated with anti-integrin α5 antibody clone IIA1 (BD Biosciences) or clone P1D6 (Millipore) at a concentration of 5 μg/ml or vehicle for 30 min. Without washing out the antibody, leukemia cells were injected into the tail vein after sublethal irradiation with 250cG from a cesium^137^ source. Twenty-four hours later, the number of human cells in the peripheral blood, bone marrow, and spleen was analyzed by flow cytometry after animals were euthanized. Human cells were recognized by flow cytometry using anti-human CD45 antibody conjugated to APC.

#### Studying the effect of echistatin pretreatment on leukemia development

Before injection, SUP-LUC2 cells were resuspended in PBS buffer at a concentration of 25 × 10^6^/ml and incubated with 62.5 μg/ml Echistatin (a potent non-specific inhibitor of integrin/ligand binding, blocking αIIβ3, αvβ3, and α5β1 integrin) or vehicle for 1 h. Echistatin was washed out by resuspension in the same concentration of normal saline plus 0.5% albumin before injection. NSG mice were sublethally irradiated with 250cG from a cesium^137^ source. Each animal was injected with SUP-LUC2 cells by tail vein within 6 h after irradiation. Starting from 1 week after leukemia cells injection, animals were injected with d-luciferin. Animals were imaged weekly with Xenogen IVIS Imaging System 200 Series (IVIS, Xenogen) and animals receiving cells pretreated with echistatin were compared to those treated with vehicle.

#### Studying the effect of knocking down integrin α5 on leukemia development

After stable clone selection, SUP-LUC2 α5 knock-down clone number 10 (named SUP-LUC2 α5–10) was chosen for animal engraftment study. We irradiated NOD/SCID mice and injected them with 5 × 10^6^ leukemia cells. Weekly imaging started 2 weeks after injection and continued for 8 weeks.

#### Studying the effect of the FAK inhibitor TAE226 on leukemia cell engraftment

NOD/SCID gamma mice were sublethally irradiated as before. Each animal was injected with 5 × 10^6^ in SUP-LUC2 cells by tail vein in 200 μl normal saline plus 0.5% human albumin within 6 h after irradiation. Then animals were divided into four groups. Control animals were treated with vehicle methylcellulose. TAE226 group animals were treated with 30 mg/kg TAE226 diluted in 200 μl methylcellulose by oral gavage. In TKI group, animals were treated with nilotinib obtained from Novartis Oncology at a dose of 20 mg/kg in 100 μl vehicle by oral gavage. Nilotinib was suspended in 0.5% hydroxypropylmethylcellulose aqueous solution containing 0.05% Tween 80 at a concentration of 4 mg/ml. Combination group animals were treated with TAE226 and nilotinib at the same concentration as previous group. Treatment commenced 2 weeks after leukemia cells were injected. Animals received treatment 5 days per week with 2 days off for total of 2 weeks. Imaging was started 2 weeks after leukemia cell injection and then weekly.

#### *In vivo* imaging

For non-invasive imaging of SUP-LUC2 cells, anesthetized mice were injected with 150 mg/kg of d-luciferin (potassium salt, Xenogen Corp., Alameda) intraperitoneally 10–15 min before imaging and were imaged using the Xenogen IVIS Imaging System 200 Series with total imaging time of 2 min. Total body bioluminescence was quantified as described (13).

Mice were sacrificed when they became moribund or unable to obtain food or water or if they lost >20% of their body weight.

### Statistical analysis

Results are expressed as means (SD) or means ± SD of three separate replicate experiments unless otherwise indicated. Levels of significance were evaluated by a two-tailed paired Student’s *t*-test, and *p* < 0.05 was considered significant.

## Results

### Integrin α5β1 expression was increased in Ph+ leukemia cells after serum starvation

**Figure 1:**
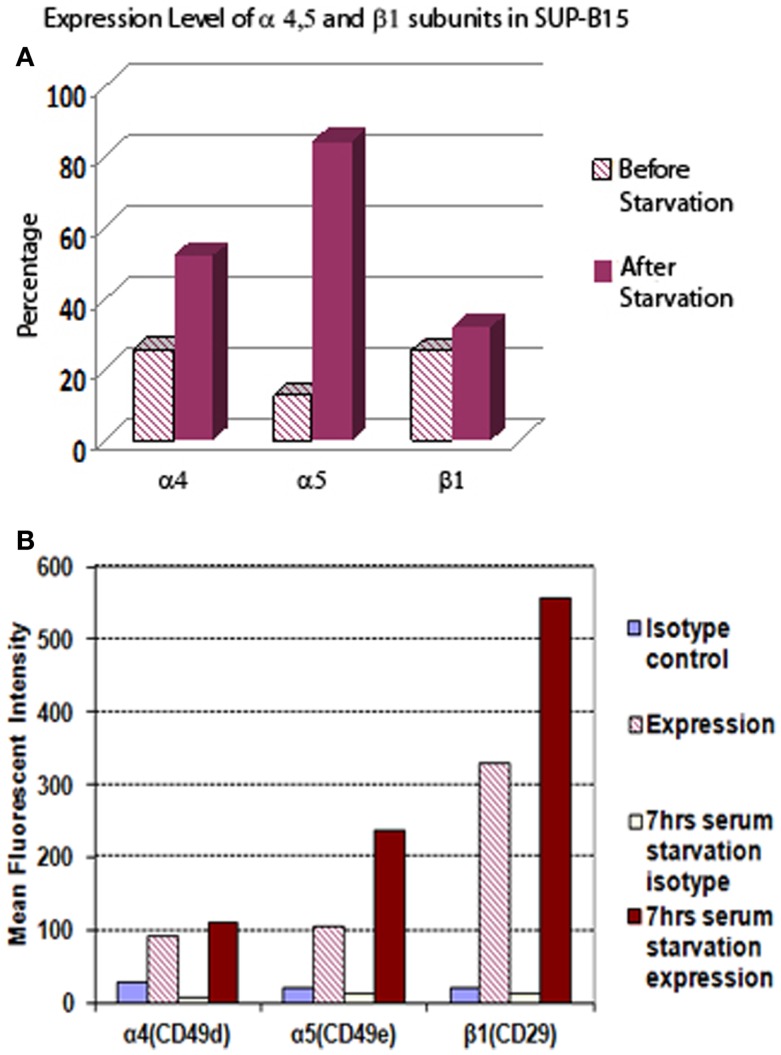
**The expression level of integrin α5 subunit in Ph+ leukemia cell line SUP-B15 is significantly increased after serum starvation**. **(A)** The percentages of cells expressing integrin α4, α5, and β1 subunits before and after serum starvation for 7 h detected by flow cytometry. **(B)** The mean fluorescent intensity (MFI) of integrin α4, α5, and β1 subunits from whole alive cell population before and after serum starvation for 7 h detected by flow cytometry.

Leukemia cells can adapt to adverse environments, such as deprivation of nutrients or oxygen, resulting in aggressive growth. In Figure 1, we show that Ph+ leukemia cells increased the expression level of integrin α5β1 under conditions of serum starvation. The percentage of cells expressing the α5 subunit increased from 12.5% before to 84.5% after serum starvation. The mean fluorescent intensity (MFI) for the α5 subunit also increased from 106 before to 237 arbitrary units of fluorescence (a.u.) after serum starvation. The percentage of cells expressing the β1 subunit did not increase in response to serum starvation (25.3 before vs. 32. 0% after). However, MFI increased significantly after serum starvation from 329 to 556 a.u. The percentage of cells expressing the α4 subunit increased from 25.6 to 52.2% and MFI from 91 to 110 after serum starvation.

### Treatment with the integrin α5 subunit antibody inhibited the adhesion of Ph+ leukemia cells to human fibronectin, and enhanced the killing of imatinib

**Figure 2:**
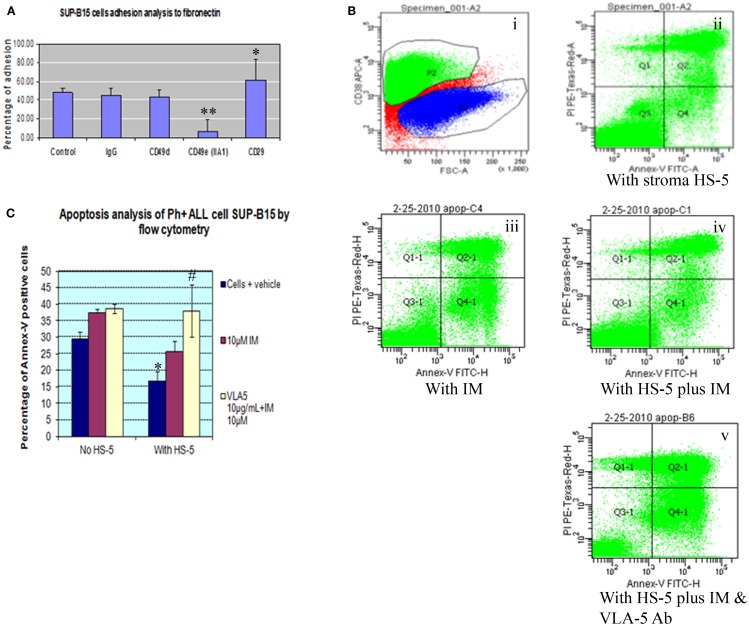
**Integrin α5 subunit antibody inhibits the adhesion of Ph+ leukemia cells to human fibronectin and enhances the killing of imatinib**. **(A)** Cell adhesion assay showed that α5 subunit inhibitory antibody (CD49e, clone IIA1 BD Biosciences) was the only tested integrin antibody that significantly inhibited the adhesion of Ph+ leukemia cells to fibronectin with adhesion percentage of 6.6 ± 3.8% compared with control IgG of 44.8 ± 7.9%, *p* < 0.01. **(B)**. Annexin-V plus PI by flow cytometry. (i) P2 gate identifies the CD38 positive cell population representing SUP-B15 Ph+ leukemia cells. (ii) Ph+ leukemia cells grown on HS-5 stromal cells. (iii) Ph+ leukemia cells treated with 10 μM imatinib (IM). (iv) SUP-B15 cultured with stromal cells treated with 10 μM imatinib. (v) SUP-B15 cultured with stromal cells treated with 10 μM imatinib and 10 μg/ml anti-α5 Ab. **(C)** Quantitative analysis for apoptosis rate of different conditions. *After culture on HS-5 cells for 24 h, the apoptosis rate of leukemia cells SUP-B15 decreased from 29.4 ± 2.3 to 16.7 ± 3%, *p* < 0.05. ^#^When inhibitory antibody to integrin α5 was combined with imatinib to treat Ph+ leukemia cells cultured on stromal cells, the apoptosis rate 38.0 ± 8.0% was significantly increased compared with imatinib by itself 25.7 ± 3.3%, *p* < 0.05.

Integrin is expressed by mouse and human long-term repopulating hematopoietic cells. In normal long-term repopulating hematopoietic cells, integrin signaling that results from α5β1 integrin binding to fibronectin protects these cells from apoptosis. To better understand the role of different integrin subunits on Ph+ leukemia cells interaction with fibronectin, we used a cell adhesion assay to check which subunit most sensitively affected the interaction of Ph+ leukemia cells with fibronectin. Result showed that the α5 subunit inhibitory antibody (CD49e) was the only one that significantly inhibited the adhesion of Ph+ leukemia cells to fibronectin with adhesion percentage of 6.6 ± 3.8 compared with control IgG 44.8 ± 7.9, *p* < 0.01 (Figure 2A). Interestingly, we found that antibody to CD29 increased the adhesion rate of Ph+ leukemia cells to fibronectin, with 61.6 ± 22.1% binding vs. 44.8 ± 7.9% of control cells, *p* < 0.05.

To investigate whether bone marrow stromal cells protected Ph+ leukemia cells from imatinib induced apoptosis, we cultured Ph+ ALL cells on the HS-5 human bone marrow stromal cell line. Cells were grown in the presence or absence of imatinib and the integrin α5 inhibitory antibody. Cells cultured with imatinib or HS-5 alone served as controls. We identified Ph+ leukemia cells using the human CD38 antibody and analyzed apoptosis with Annexin-V combined with propidium iodide by flow cytometry. As shown in Figures 2B,C, after culture on HS-5 cells for 24 h, the apoptosis rate of leukemia cells SUP-B15 decreased from 29.4 ± 2.3 to 16.7 ± 3%, *p* < 0.05. HS-5 combined with imatinib decreased apoptosis rate from 37.4 ± 1.2% without HS-5 to 25.7 ± 3.3%, but this result was not statistically significant, *p* = 0.45. When inhibitory antibody to integrin α5 was combined with imatinib to treat Ph+ leukemia cells cultured on stromal cells, the apoptosis rate 38.0 ± 8.0% significantly increased compared with imatinib by itself 25.7 ± 3.3%, *p* < 0.05. There was no significant difference for the leukemia apoptosis rate between cells cultured with and without HS-5 cells when α5 antibody was included (38.0 ± 8.0 vs. 38.6 ± 1.4, *p* > 0.05). This confirmed that the protective effect of stromal cells was mediated through the α5 integrin, and demonstrated the synergistic effect of integrin α5 antibody with imatinib.

### Blocking integrin α5 reduced the engraftment of Ph+ leukemia cells in immunodeficient mice

**Figure 3:**
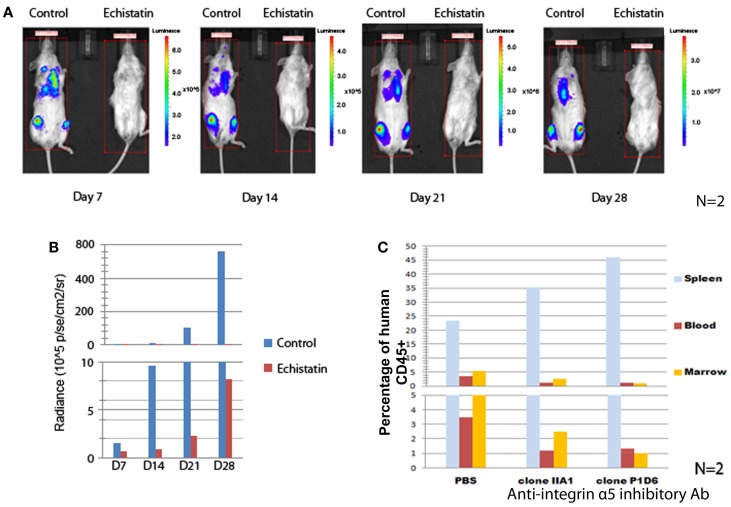
**Blocking integrin α5 affects the engraftment of Ph+ leukemia cells in immunodeficient mice**. **(A)** The incubation for 1 h of Ph+ leukemia cells with disintegrin, a peptide inhibitor of integrins, impaired the engraftment of leukemia in NSG mice. Representative figures of *n* = 2 showed bioluminescence imaging from day 7 to 28 after inoculation of leukemia cells. **(B)** Animal total body bioluminescence was measured using the Xenogen IVIS Imaging System 200 Series with total imaging time of 2 min and compared with control animals that received cells that were not treated with Echistatin. **(C)** Anti-integrin α5 inhibitory antibodies clone IIA1 (BD Biosciences) and clone P1D6 (Millipore) decreased the engraftment of Ph+ leukemia cells in the bone marrow of NOD/SCID mice (*n* = 2).

To demonstrate the role of integrins in enhancing the survival of Ph+ leukemia cells *in vivo*, we first performed experiments to study the effect of integrin inhibition on short term Ph+ leukemia cell engraftment. Echistatin is a potent non-specific inhibitor of integrin/ligand binding, blocking αIIβ3, αvβ3, and α5β1 integrin (14, 15). We used echistatin to test if blocking integrin α5β1 integrin could affect the engraftment of Ph+ leukemia cells in immunodeficient mice. As shown in Figures 3A,B, after incubation with echistatin for about 30 min, the engraftment was significantly delayed from day 7 to 1 month post injection. Echistatin did not significantly affect the viability of injected cells (data not shown).

To understand how specifically integrin α5 affects the *in vivo* effect, we incubated the Ph+ leukemia cells with two types of integrin α5 inhibitory antibodies. We then injected leukemia cells into NOD/SCID mice to observe the effect on short term engraftment. Twenty-four hours after injection, mice (*n* = 2) were sacrificed and peripheral blood, spleen, and bone marrow samples were obtained and analyzed by flow cytometry. We found that incubating leukemia cells with antibodies that block α5 integrin decreased the engraftment percentage from 5.3 to 1–2% compared with isotype control in the bone marrow, and from 3.5 to 1.2% in the peripheral blood (Figure 3C). Most of the leukemia cells were sequestered in the spleen. The percentage of leukemia cells increased from 23.4% when incubated with an isotype control to 35.2 or 45.9% with two different clones of integrin α5 antibodies.

### Knocking down integrin α5 delayed the long-term engraftment and progression of Ph+ leukemia cells in immunodeficient mice

**Figure 4:**
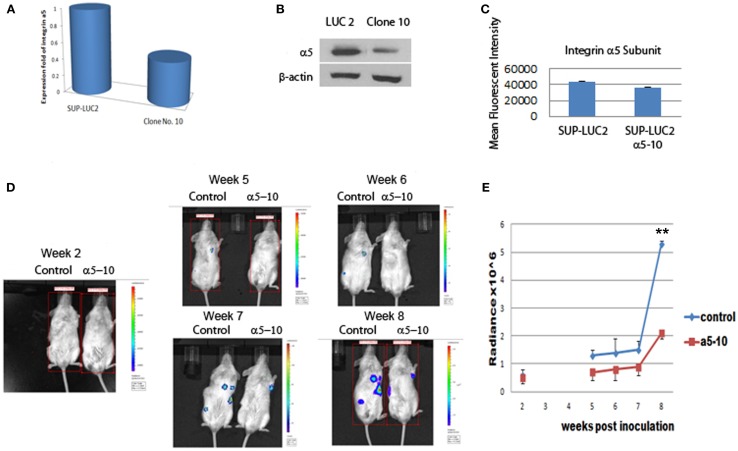
**Knocking down integrin α5 delayed the engraftment of Ph+ leukemia cells in immunodeficient NOD/SCID mice**. **(A)** Real-time QPCR assay showed that we had successfully knocked down the expression level of the integrin α5 knocking in clone 10. The cycle threshold (Ct) of the target gene (integrin α5) was normalized to the chosen reference gene GAPHD. Relative quantification, *R* = 2^−(ΔCt sample − ΔCt control)^. Result shows the fold change normalized to SUP-LUC2 cells. **(B)** Western blot showed a reduced protein expression level of integrin α5 in clone 10. **(C)** Flow cytometry showed reduced mean fluorescent intensity of integrin α5 in clone 10. **(D)** Representative figures showed bioluminescent imaging at 2 weeks, and 5–8 weeks post leukemia cells inoculation. **(E)** Animal total body bioluminescence was quantified to compare with control. **The levels of bioluminescence became most significantly different 2 months post inoculation between the α5 knock-down group and control group [mean (SD) radiance vs. control, 5.3 (0.1) vs. 2.1 (0.2) × 10^6^ p/s/cm^2^/sr, *p* < 0.01 at 2 months, *n* = 3.].

To further test the role of integrin α5 on the long-term engraftment of Ph+ cells in the bone marrow, the integrin α5 subunit was knocked down by human integrin α5 shRNA lentiviral particles. We selected the Ph+ leukemia cell clone (clone 10) with the highest degree of knock-down of integrin α5 by real-time quantitative PCR assay (Figure 4A). Western blot confirmed reduction of integrin α5 protein expression (Figure 4B). Flow cytometry also demonstrated reduced MFI (Figure 4C).

We used luciferase to monitor the engraftment level of Ph+ leukemia cells with and without knock-down of the α5 integrin *in vivo* in NOD/SCID mice. Five weeks post inoculation, the levels of bioluminescence in mice receiving Ph+ leukemia cells with integrin α5 knock-down were significantly lower than in the control group [mean (SD) radiance vs. control, 0.7 (0.3) vs. 1.3 (0.2) × 10^6^ p/s/cm^2^/sr, *p* < 0.05 on day 37]. Since NOD/SCID mice have some NK cell and macrophage function, the xenografted human leukemia cell percentage is lower in NOS/SCID mice than that in NSG mice. Our experiments also confirmed this phenomenon. It also took longer time to show the engraftment differences between integrin knock-down group and control group when using NOS/SCID mice. The levels of bioluminescence became most significantly different 2 months post inoculation between the α5 knock-down group and the control group [mean (SD) radiance vs. control, 5.3 (0.1) vs. 2.1 (0.2) × 10^6^ p/s/cm^2^/sr, *p* < 0.01 at 2 months] (Figures 4D,E).

### The FAK inhibitor TAE226 inhibited Ph+ lymphoblastic leukemia proliferation *in vitro*

**Figure 5:**
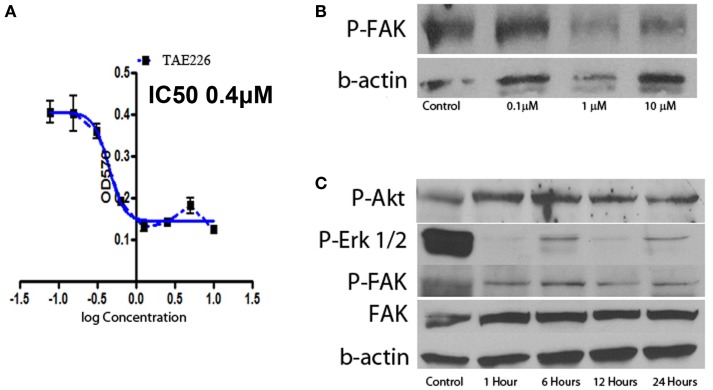
**Effect of FAK inhibitors on Ph+ ALL**. **(A)** TAE226 inhibits SUP-B15 cell growth with an IC50 0.4 μM. **(B)** Western blot with protein obtained from SUP-B15 cells, which were cultured with different concentration of TAE226 from 0 to 10 μM for 1 h. **(C)** Western blot with protein obtained from SUP-B15 cells, which were cultured with 1 μM TAE226 from 1 to 24 h. **(B,C)** Western blot showed that TAE226 reduced phosphorylation of FAK and AKT, ERK1/2, which are downstream in the FAK signaling pathway.

Focal adhesion kinase is the downstream signal of integrin α5β1. TAE226 is a novel small molecule ATP competitive inhibitor of FAK and tyrosine kinase activity of insulin-like growth factor I (16, 17). Here we used serial dilution of TAE226 to observe how it affected the leukemia cell proliferation. TAE226 significantly inhibited leukemia cell proliferation at concentrations above 75 nM. Figure 5A shows the dose–response curves of effects of TAE226 on Ph+ ALL leukemia growth with an IC 50 dosage of 0.26 μM. To explore which signaling pathway is involved in TAE226-induced apoptosis, we dissected out downstream molecules of FAK by Western blot. TAE226 inhibited phosphorylation of FAK in a dose- and time-dependent manner in Ph+ leukemia cells. Erk1/2 activation was also suppressed, whereas the inhibition of Akt activity was modest (Figures 5B,C).

### FAK inhibitor TAE226 synergized with nilotinib to block Ph+ lymphoblastic leukemia growth *in vivo*

**Figure 6:**
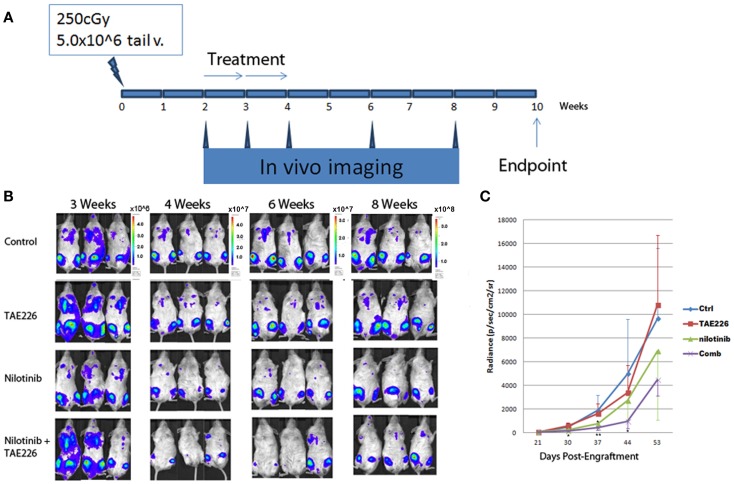
**TAE226 reduced Ph+ lymphoblastic leukemia progression in NSG mice**. **(A)** Animal experiment scheme. **(B)** Animal bioluminescent imaging at 3–8 weeks after inoculation of leukemia cells. **(C)** Animal total body bioluminescence was quantified for comparison (*N* = 4 ~ 6). *On day 30 and 44 post inoculation, mean radiance (SD) in animals receiving nilotinib in combination with TAE226 was significantly lower than that in the control group, *p* < 0.05. On day 37 post inoculation, animals receiving nilotinib alone only showed significant differences compared with the control group, *p* = 0.01. **On day 37 post inoculation, levels of bioluminescence in the combination treatment group were most significantly reduced compared with the control group [418 (221) vs. 1908 (1281) × 10^6^ p/s/cm^2^/sr, *p* < 0.01].

We confirmed a potent inhibitory effect of TAE226 on Ph+ leukemia cell growth *in vitro*. For the next step, we used immunocompromised NSG mice to generate human leukemia xenografts. The animals were treated with an oral administration of either TAE226 (30 mg/kg), TKI nilotinib, the combination of TAE226 and nilotinib or methylcellulose as vehicle control. The daily administration started 2 weeks after inoculation and continued for 2 weeks (days 1–14 with 2 days break each week). Results in Figure 6 showed that even though treatment with FAK inhibitor TAE226 alone did not significantly inhibit leukemia growth in mice by body bioluminescence [mean (SD) radiance vs. control, 3386 (2294) vs. 4979 (4609) × 10^6^ p/s/cm^2^/sr, *p* = 0.38 on day 44], the FAK inhibitor TAE226 in combination with TKI nilotinib provided the most optimum growth inhibition 4–6 weeks post inoculation. On day 30 post inoculation, mean radiance (SD) in animals receiving nilotinib in combination with TAE226 was significantly lower than that in the control group [160 (121) vs. 498 (306) × 10^6^ p/s/cm^2^/sr, *p* = 0.02]. On day 37 post inoculation, levels of bioluminescence in the combination treatment group were most significantly reduced compared with the control group [418 (221) vs. 1908 (1281) × 10^6^ p/s/cm^2^/sr, *p* < 0.01]. There was still a significant difference on day 44 with animals from the control group [938 vs. 4979 × 10^6^ p/s/cm^2^/sr, *p* = 0.03]. Animals receiving nilotinib alone only showed significant differences on day 37 post inoculation compared with the control group, with mean radiance (SD) [752 (391) vs. 1908 (1281), *p* = 0.01].

## Discussion

In this study, we report that integrin α5β1 and its downstream signal FAK may be good targets for developing new strategies to treat Ph+ ALL.

### Integrin α5β1 is a specific target for Ph+ ALL

Through interaction of integrin α5β1 (VLA-5) with fibronectin, leukemia cells adhere to marrow stroma and become quiescent and resistant to chemotherapy (18, 19). Acute lymphoblastic leukemia patients with Ph+ have been shown to express very high levels of both VLA-4 and VLA-5 (3). Introduction of BCR/ABL into hematopoietic cell lines enhances adhesion through VLA-5 by inside-out signal (4). Our results in Figure 1 showed that both the percentage and intensity of integrin α5 subunit increased significantly more than the α4 subunit in Ph+ ALL cells after serum starvation. Upregulation of VLA-5 under conditions of stress may be one of the reasons Ph+ leukemia has been difficult to treat. The upregulation of integrin α5β1 can protect leukemia cells from apoptosis and provide a growth advantage relative to normal hematopoietic cells. For instance, it has been shown that α5β1 integrin upregulated the resistance to TNF induced apoptosis in adherent leukemia cells (5).

van der Loo and colleagues have shown that mouse long-term *in vivo* repopulating stem cells and primitive human NOD/SCID mouse repopulating cells, bind to the extracellular matrix protein fibronectin through VLA-5 *in vitro*. This binding is specific and can be inhibited by antibodies to VLA-5 (20). In Figure 2 we show that α5 subunit antibody specifically blocked the adhesion of Ph+ ALL cells to fibronectin. This indicates that integrin α5β1 is specific to Ph+ ALL cells as well as in the normal hematopoietic stem/progenitor cells and could be an important treatment target for this type leukemia. So targeting α5β1 might be able to block the Ph+ leukemia cells adhesion to bone marrow stroma fibronectin and induce apoptosis when combined with TKIs. However, off target effects on hematopoietic stem cells could be a problem and would have to be considered in developing such a strategy.

In order to confirm the specific protective effect of integrin α5β1 signaling through interaction with FN for Ph+ leukemia cells, we cultured SUP-B15 on the human bone marrow stromal cell line HS-5. We found that the apoptosis rate of SUP-B15 cells decreased when cultured on stroma. Integrin α5 inhibitory antibody blocked the protective effect of stromal cells and synergized with imatinib to induce apoptosis.

As it is reviewed in Aoudjit’s article, ECM/integrin signaling protects tumor cells from drug-induced apoptosis (21). Different integrin family members might be involved in different types of tumors. For instance, the α4β1 integrin interaction with fibronectin prevents apoptosis in B cell chronic lymphocytic leukemia (22). Blocking integrin alpha4 can sensitize drug resistant of pre-B acute lymphoblastic leukemia to chemotherapy (23). The interaction between VLA-4 on acute myeloid leukemic cells and fibronectin on stromal cells is crucial in minimal residual disease and for acute myelogenous leukemia prognosis (24). The α2β1 integrin was shown to be important for integrin-mediated attachment to collagen type I during metastasis of breast cancer cells to the bone (25). α4β1 integrin was involved in increased peritoneal metastasis and αvb3 in tumor proliferation for ovarian cancer (26). Our data suggest that integrin α5β1 is a promising target for Ph+ ALL.

Integrin α5β1 (VLA-5) is the key adhesion molecule for lymphocyte homing. In order to further investigate the importance of integrin α5β1 on Ph+ ALL cells, we incubated Ph+ leukemia cells with disintegrin, a peptide inhibitor of integrins, and specific α5 inhibitory antibody, and specially knocked down α5 subunit by shRNA and then tested the Ph+ leukemia engraftment in immunodeficient mice. Using two types of immunodeficient mice, NOD/SCID and NSG, we found that every method we used to block integrin α5 impaired or delayed the engraftment of leukemia in immunodeficient mice. Although, since the engraftment levels were different between the two types of mice, it took longer for NOD/SCID mice to show the differences in engraftment since the overall engraftment levels were lower. This result indicates that integrin α5β1 (VLA-5) is important for Ph+ leukemia cells’ engraftment into the bone marrow.

Since the integrin α5β1 inhibitor is not commercially available and a large amount of integrin α5β1 antibodies are needed to be purified for *in vivo* experimental usage, we are not able to directly test the effects of integrin α5β1 inhibitor or antibodies in the treatment for Ph+ leukemia using our animal model. Developing a specific antagonist for integrin α5β1 is our next study direction. In order to test our hypothesis that blocking this pathway could improve leukemia outcome, we used TAE226, the inhibitor of FAK, an integrin downstream factor, in combination with the TKI, nilotinib, to determine whether they would synergize.

### FAK inhibitor TAE226 plays a synergistic role with nilotinib in inhibiting progression of Ph+ leukemia in xenografts

Focal adhesion kinase is a non-receptor type tyrosine kinase that is activated from integrins and FAK signaling is critical for tumor growth. FAK is constitutively activated in Ph+ ALL by the BCR–ABL translocation (10). FAK silencing inhibits leukemogenesis in BCR/ABL-transformed hematopoietic cells (D10). TAE226 is a novel low-molecular weight inhibitor of FAK. TAE226 inhibits the phosphorylation of FAK (27). We used TAE226 to study how it affects Ph+ leukemia growth *in vitro*. As shown in Figure 5, TAE226 significantly inhibited leukemia cell growth at a concentration above 75 nM with an IC 50 of 0.26 μM. Western blot showed that TAE226 inhibited phosphorylation of FAK in a dose- and time-dependent manner and suppressed Erk1/2 activation in Ph+ leukemia cells. TAE226 did not significantly inhibit the Ph+ leukemia growth at a concentration of 30 mg/kg *in vivo* in a xenografted animal model. However, when combined with the BCR–ABL TKI nilotinib, TAE226 at 30 mg/kg synergized with nilotinib to provide the most optimum growth inhibition between 4 and 6 weeks post inoculation.

TAE226 has demonstrated anti-tumor activity in experimental models in ovarian cancer, gliomas, esophageal, and breast cancer. Ozkal et al., have performed a comprehensive study of FAK expression in normal lymphoid tissues and leukemias/lymphomas by immunohistochemistry and found that FAK was present in the majority of cases of B-(75%) and absent in T-(0%) lymphoblastic leukemias/lymphomas (28). Our study supports that FAK is a good target for Ph+ ALL.

## Conflict of Interest Statement

The authors declare that the research was conducted in the absence of any commercial or financial relationships that could be construed as a potential conflict of interest.
